# Role of a critical visceral adipose tissue threshold (CVATT) in metabolic syndrome: implications for controlling dietary carbohydrates: a review

**DOI:** 10.1186/1743-7075-1-12

**Published:** 2004-11-05

**Authors:** Eric S Freedland

**Affiliations:** 1 Boston University School of Medicine, 5 Bessom Street, No. 318, Marblehead, MA 01945, USA

## Abstract

There are likely many scenarios and pathways that can lead to metabolic syndrome. This paper reviews mechanisms by which the accumulation of visceral adipose tissue (VAT) may contribute to the metabolic syndrome, and explores the paradigm of a critical VAT threshold (CVATT). Exceeding the CVATT may result in a number of metabolic disturbances such as insulin resistance to glucose uptake by cells. Metabolic profiles of patients with visceral obesity may substantially improve after only modest weight loss. This could reflect a significant reduction in the amount of VAT relative to peripheral or subcutaneous fat depots, thereby maintaining VAT below the CVATT. The CVATT may be unique for each individual. This may help explain the phenomena of apparently lean individuals with metabolic syndrome, the so-called metabolically normal weight (MONW), as well as the obese with normal metabolic profiles, i.e., metabolically normal obese (MNO), and those who are "fit and fat." The concept of CVATT may have implications for prevention and treatment of metabolic syndrome, which may include controlling dietary carbohydrates. The identification of the CVATT is admittedly difficult and its anatomical boundaries are not well-defined. Thus, the CVATT will continue to be a work in progress.

## Introduction

Arguably, the major pathogenic factor in the metabolic syndrome is central obesity [1,2]. While abdominal obesity is determined by the accumulation of both subcutaneous adipose tissue (SCAT) and visceral adipose tissue (VAT), the excess accumulation of VAT appears to play a more significant pathogenic role. VAT depots, located in the body cavity beneath the abdominal muscles, are composed of the greater and lesser omentum (peritoneum that is attached to the stomach and links it with other abdominal organs) and the mesenteric fat. A lesser amount of VAT is located retroperitoneally. In general, VAT accounts for up to 20 percent of total fat in men and 5–8 percent in women. The abdominal SCAT is located immediately beneath the skin and on top of the abdominal musculature. The predominance of lower body fat is SCAT, most of which is stored in the femoral and gluteal regions [3-5]. Abdominal obesity can reflect a predominance of flabby SCAT; a firm, only modestly enlarged waist line resulting from deep VAT pushing the abdominal musculature outward; or a combination of enlarged SCAT and VAT depots. With the advent of more precise imaging techniques, e.g., magnetic resonance imaging (MRI) [6], computed tomography (CT) [7], and ultrasound [8], it has become evident that the accumulation of VAT not only accompanies but antedates the onset of the components of the metabolic syndrome and related disorders, e.g., insulin resistance, hypertension [9], glucose intolerance [10], type 2 diabetes, and coronary heart disease [11].

To date, it has not yet been established that insulin resistance, i.e., resistance of cells to insulin's effects, is responsible for the onset of the multiple risk factors associated with insulin resistance syndrome and subsequent development of atherosclerosis and cardiac events [12]. In fact, National Cholesterol Education Program Adult Treatment Panel (ATP III) criteria for Metabolic Syndrome have been found to have a low sensitivity for predicting insulin resistance [13-15] and may be better thought of as predictors for cardiovascular risk [16]. In a recent study of a large number of apparently healthy men and women of varying age, VAT area was significantly associated with all of the metabolic syndrome criteria as defined by the NCEP ATP III. This was independent of insulin sensitivity and SCAT area. Insulin sensitivity was found to be independently associated with the criteria for HDL cholesterol, triglycerides (TGs), and fasting plasma glucose (FPG). SCAT area was independently correlated with only waist circumference after adjusting for VAT area and insulin sensitivity [11]. In addition, the study results showed that clinical assessments of increased waist size and TG levels are strongly associated with decreased insulin sensitivity and increased VAT in individuals with fasting FPG <6.4 mmol/L [11].

The term "metabolic syndrome" is now preferable to "insulin resistance syndrome," and has a prevalence of 25 percent in U.S. individuals age >20, rising to >40 percent by age 60 [17]. The importance of central obesity is well-recognized in the definitions of metabolic syndrome [18] per the American College of Endocrinology, [19,20] National Cholesterol Education Program Adult Treatment Panel (ATP III), [21] European Group for the Study of Insulin Resistance, [22] and World Health Organization (WHO) [23]. However, even apparently lean individuals with normal BMIs can have a significant accumulation of VAT with increased risk factors for cardiovascular disease and diabetes (metabolically obese normal weight; MONW) [24-26]. Meanwhile, obese individuals with large BMIs but relatively little VAT can present with normal metabolic profiles and a paucity of risk factors for metabolic syndrome, cardiovascular disease, and diabetes, i.e., the metabolically normal obese; MNO) [5,27].

### Ectopic Fat Storage Syndrome

The *ectopic fat storage syndrome *hypothesis suggests that as adipocytes hypertrophy and reach their capacity for storing more fat, then additional fat from excess dietary lipids or calories is deferred to non-adipose tissues intracellularly, e.g. liver, skeletal muscle, heart, and the beta cells of the pancreas where they can exert toxic effects and dysfunction [7]. This "lipotoxicity" may also be exacerbated by impaired oxidation of fat within tissues [7,28-30]. Furthermore, adipose tissue is a major endocrine organ that secretes numerous polypeptide hormones and cytokines that are proinflammatory and proatherogenic. These play a major role in affecting insulin action in skeletal muscle and creating a low-grade state of inflammation and endothelial dysfunction [31]. Compared to SCAT, VAT has been correlated more with endothelial dysfunction [32,33].

### CVATT – A Working Hypothesis

**Figure 1:**
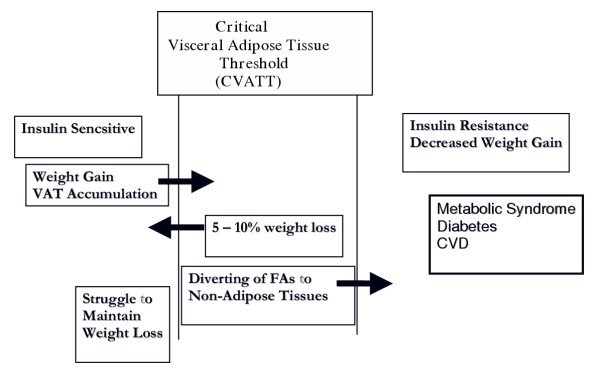
**Critical Visceral Adipose Tissue Threshold (CVATT). **According to the hypothesis, there is an individual range for accumulating a critical amount of visceral adipose tissue (VAT). Insulin sensitivity is important for weight gain and accumulation of VAT. Once the critical VAT threshold (CVATT) is reached, insulin resistance occurs, which may be protective initially and impair further weight and fat gain. Continuation of VAT accumulation can lead to metabolic syndrome. However, only a modest weight loss (5–10 percent) with accompanying VAT loss can reverse the process.

It must be emphasized that the current proposal is a working hypothesis. Figure 1 describes a critical VAT threshold (CVATT) which is unique for a given individual and represents a range for the accumulation of a critical mass of VAT (CVATT) that when achieved, leads to the development of metabolic syndrome. Note that insulin sensitivity is important for weight gain [34] and accumulation of VAT, and investigators have proposed that insulin resistance may actually, to a certain extent, be beneficial by protecting cells with already impaired fatty acid oxidation. Once the CVATT is reached, insulin resistance (IR) occurs, which may be protective initially [29,35-37]. In addition to protecting against further weight and fat gain [34,38-41], insulin resistance prevents glucose and more fat from entering the cell and becoming preferentially oxidized. Hence, insulin resistance also allows intracellular fat already present within the cell to become oxidized rather than cause further damage through "lipotoxicity [29,30,40,42,43]."

### Implications of VAT loss

It is encouraging that only a modest loss of 5–10 percent of body weight in obese patients is associated with preferential mobilization of VAT compared to SCAT, leading to simultaneous improvement in all metabolic markers of CHD risk. Such modest weight loss can prevent and reverse type 2 diabetes [44-48], and sustained weight loss in obese women results in a reduction in elevated inflammatory cytokine levels and an amelioration of endothelial dysfunction [49,50]. Surgical removal of VAT may reduce insulin resistance and plasma insulin levels [51,52], while liposuction of SCAT does not confer metabolic benefits [53]. Weight loss usually leads to VAT reduction as well as reduction of depots of fat in non-adipose organs, thereby improving insulin sensitivity [48]. However, once individuals improve insulin sensitivity by losing weight and crossing beneath their CVATT [48], they may now be more susceptible to weight gain and struggle to maintain this new state.

With total weight loss, those with greater amounts of VAT initially lose more VAT, and VAT is more sensitive to weight reduction because the VAT adipocyte is more metabolically active and sensitive to lipolysis [5,54]. After the initial weight loss, further dietary restriction may lead to an overall reduction in body fat, rather than specific loss from a particular site. The metabolic improvements observed with only modest reductions in total weight underscore the importance of VAT in insulin resistance and metabolic abnormalities [48,55]. Once the individual has lost a significant amount of VAT and is now below his CVATT, improvement in insulin sensitivity does not bear a linear relationship to the magnitude of weight loss [48].

The identification of the CVATT is admittedly difficult and its anatomical boundaries are not well-defined. Thus, the CVATT will continue to be a work in progress. While there are numerous studies linking VAT quantity to insulin resistance and metabolic syndrome, this does not necessarily prove that VAT is the cause. However, there are a number of plausible mechanisms linking VAT to the metabolic syndrome.

### Adipose tissue as an endocrine organ

#### Adipokines

Once thought to be an inert energy storage depot, adipose tissue is now known to be a critical endocrine organ. The term "adipocytokines" or "adipokines" has been used to describe the numerous adipocyte secretory products which include: adiponectin, adipsin, estrogen, angiotensin II, angiotensinogen, leptin, plasminogen activator I (PAI-1), agouti protein, resistin [56], acylation stimulating protein (ASP), bone morphogenic protein (BMP), prostaglandins, IGF-1, and various IGF binding proteins, tumor necrosis factor alpha (TNFα), interleukins (ILs), transforming growth factor (TGF)-B [57], and fibroblasts, as well as FFAs themselves. Adipokines such as IL-6 and PAI-1 are more highly secreted by VAT than abdominal SCAT [58,59], while leptin is more highly secreted by SCAT [60]. Adipokines from VAT can be delivered via the portal system directly to the liver where they can affect hepatic, and ultimately systemic, inflammation. In an ex vivo study, VAT released greater amount of IL-6 and PAI-1 compared with abdominal SCAT [58,61].

Adiponectin has many beneficial vascular and metabolic effects, e.g., it serves as an anti-inflammatory molecule for vascular walls as well as adipose tissue, inhibits vascular smooth muscle proliferation, protects endothelium from macrophage adhesion and macrophage-induced injury [62], may increase fatty acid oxidation in peripheral tissues [63], protects against ectopic fat storage and has been linked with insulin sensitivity [64,65].

Ironically, although produced by adipose tissue, adiponectin levels are lowered with greater degrees of obesity and with overfeeding. Decreased concentrations of adiponectin are associated with type 2 diabetes, hypertension, elevated glucose levels, insulin and TGs, and cardiovascular disease (CVD). It has been suggested that adiponectin is under feedback inhibition in obesity and reduced in patients with metabolic syndrome [66]. Adiponectin mRNA and protein levels have been found to be reduced in omental VAT compared with SCAT [67], and VAT may also produce an as-yet-identified factor that destabilizes adiponectin mRNA [66,68]. The strong inverse correlation between serum adiponectin levels and VAT mass may in part explain the link between VAT and metabolic syndrome [66]. Over 90 percent of the adipokines released by adipose tissue, except for adiponectin and leptin, could be attributed to non-fat cells, e.g., macrophages, retained in the adipose tissue matrix [61].

### Implications of fat mass expansion

Fat mass can expand in one of two ways: individual adipocytes can increase in volume or they can increase in number as more are derived from preadipocytes. As adipocytes grow larger, they become dysfunctional. The total number of adipocytes is increased with increasing fat mass, but it is the increased number and percentage of large adipocytes, compared to the smaller ones, that may partially account for the inability of adipose tissue to function properly [69]. While the smaller adipose cells tend to be more insulin sensitive, large adipocytes become insulin resistant and contribute more to the metabolic problems associated with obesity [69].

Preadipocytes from the SCAT depots have a greater differentiation capacity than those from the VAT depots [70,71]. The differentiation of preadipocytes into lipid-storing adipocytes is regulated in part by the nuclear hormone receptor, peroxisome proliferators activated receptor (PPAR). Activation of this receptor by natural ligands, such as prostaglandin metabolites, or synthetic ligands, such as thiazolidinediones (TZDs), leads to stimulation of the differentiation pathway [71]. This increases the number of smaller adipocytes in SCAT with a high avidity for FA and TG uptake. These increased adipose stores made up of new, smaller, more insulin sensitive adipocytes act as a sink or powerful 'buffers,' avidly absorbing circulating fatty acids and triglycerides in the postprandial period. This prevents their diversion to non-adipose tissues, thereby protecting against ectopic fat syndrome and metabolic syndrome. It has been proposed that an inability to differentiate new adipocytes to accommodate and store excess energy, underlies the development of type 2 diabetes [72,73].

#### A thiazolidinedione (TZD) paradox

TZDs can increase the number of new fat cells, and because obesity is a major cause of insulin resistance, this represents an apparent paradox. Ex-vivo studies of human preadipocytes from SCAT and VAT depots have demonstrated that TZD-stimulated differentiation is much greater in SCAT than VAT preadipocytes [71]. Since TZDs selectively promote adipogenesis in SCAT and not VAT, this would encourage the redistribution of body fat away from "harmful" VAT sites and toward "safer" SCAT ones [74-76]. Thus, in this way, TZDs could allow for pushing the patient to below his CVATT. Paradoxically, the TZDs can lead to weight gain while improving insulin sensitivity as the new SCAT adipocytes continue to trap FA and as fat storage continues, eventually the new adipocytes will enlarge, become less insulin sensitive, and ultimately contribute to insulin resistance [77]. TZDs may also exert anti-inflammatory effects on adipocytes by reducing the production of serum amyloid A (SAA) and preventing the TNFα-mediated expression of adiponectin production [69].

#### Adipose macrophages

Macrophages increase their accumulation within fat depots in direct proportion to increases in adipose tissue and adipocyte size. The increased macrophage activity observed in the adipose tissue of the obese may reflect a combination of conversion of local preadipocytes to macrophages and activation and recruitment of resident macrophages and circulating monocytes. This seems to occur after the onset of adiposity but prior to insulin resistance, and supports the notion that pathophysiological consequences of obesity involve macrophages and inflammation that contribute to insulin resistance and metabolic syndrome [78,79]. Evidence suggests that macrophages and adipocytes not only express overlapping sets of genes and serve similar functions, but also commingle in the same part of the body – the fat tissue [80].

### VAT Versus SCAT (See figure 2)

**Figure 2:**
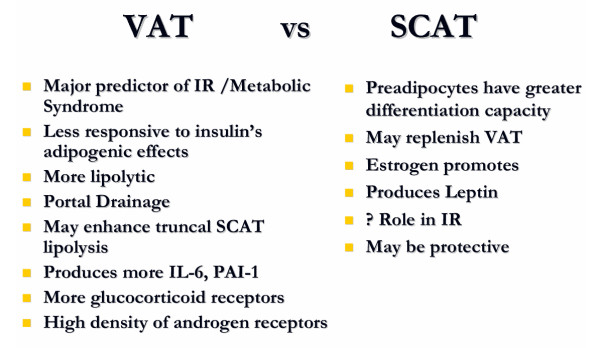
VAT versus SCAT

#### VAT

There are numerous inherent differences between VAT and SCAT. VAT is a major predictor for insulin resistance [81] and metabolic syndrome [11]. Compared to SCAT, VAT adipocytes have a higher rate of lipolysis, which is more readily stimulated by catecholamines and less readily suppressed by insulin [82]. VAT also produces more IL-6 and plasminogen activator inhibitor-1 (PAI-1) [81]. The "Portal Theory" suggests that insulin resistance and many of its related features could arise from VAT delivering free fatty acids (FFAs) at a high rate to the liver via the portal vein into which VAT directly drains [83,84]. This, in turn, would increase hepatic glucose production, reduce hepatic insulin clearance, and ultimately lead to insulin resistance, hyperinsulinemia, hyperglycemia as well as non-alcoholic fatty liver disease (NAFLD). FFA flux could also lead to enhanced production of triglycerides (TGs) and apolipoprotein B-rich lipoproteins, which are features of the insulin resistance syndrome [55,85]. Delivery of VAT derived pro-inflammatory cytokines may contribute to hepatic pathology such as non-alcoholic steatohepatitis (NASH). VAT also releases a large amount of glycerol which enters the liver where it can be converted to glucose, thereby contributing to hyperglycemia [86]. It is likely that the relationship observed between VAT and metabolic complications may not exclusively result from FFA flux from VAT into the portal vein and the portal theory does not adequately hold up as the sole explanation of the role of VAT in metabolic syndrome [7].

#### VAT has twice the glucose uptake rate as SCAT

Recently, omental VAT cells have been shown to have an approximately two-fold higher rate of insulin-stimulated glucose uptake compared with SCAT adipocytes, and this could be explained by a higher GLUT-4 expression [87]. Perhaps in situations with a high intake of dietary glycemic load, a higher rate of glucose uptake and subsequently lipogenesis might be one mechanism by which TGs are stored preferentially in the VAT depot. VAT is highly lipolytic and resistant to insulin's lipogenic effects yet apparently can remain insulin sensitive to glucose uptake. This efficiency in glucose uptake may reflect VAT's ability to accumulate and maintain its activity. Enhanced glucose utilization in VAT would be accompanied by less lipid oxidation, which would indirectly promote TG storage [87].

#### Testosterone

VAT has a high density of androgen receptors and testosterone which can amplify its own effect by up-regulation of androgen receptors, inhibiting the expression of lipoprotein lipase (LPL) and FA uptake [5,88]. In men, VAT is strongly negatively correlated with plasma total and free testosterone and sex-hormone binding globulin (SHBG) concentrations. Thus, in young men whose plasma total testosterone and free testosterone are high, the amount of VAT is low. As men age, exceed their 20s, and reach middle age, their total and free testosterone decline, more fat is deposited in VAT stores, they often develop the "pot belly," and their risk for CHD increases [5,89]. The effects of testosterone on insulin resistance and metabolic syndrome risk factors are opposite in men and women [5,88,90,91]. Testosterone production often declines in women as they age, but VAT obesity in women is associated with elevated levels of total testosterone, free testosterone., and SHBG [92]. Hyperandrogenicity can also occur in polycystic ovary syndrome, where hyperinsulinemia can stimulate ovarian androgen production and suppress serum SHBG [88,93]. While weight loss in both sexes has been consistently shown to reverse the abnormalities in testosterone levels [94-97], a number of placebo controlled studies have consistently demonstrated that administering testosterone to obese men resulted in a significant reduction in VAT. This occurred without significantly altering amounts of total body fat or lean body mass [89,98-100]. However, the use of testosterone for VAT obesity is left open to debate [90].

#### 11-β-Hydroxydehydrogenase1 (11-β HSD1)

Patients with type 2 diabetes and metabolic syndrome often appear Cushingoid, yet they invariably do not have elevated plasma cortisol [101]. Compared to SCAT, VAT has more glucocorticoid receptors [88]. The enzyme 11-β hydroxysteroid dehydrogenase type 1 (11-β HSD1) converts inactive cortisone to the active compound cortisol, and, if overexpressed, may cause increases in local cortisol concentrations [101]. Local production of active cortisol from inactive cortisone driven by 11-β-HSD-1 activity is very high in VAT and barely detectable in SCAT. Therefore it is likely that the VAT depot actively contributes to the production of high local concentrations of cortisol, which might not be reflected by plasma levels. These, in turn, might contribute to an increase in VAT accumulation [102]. 11-βHSD1 inhibition holds promise as a therapeutic target for VAT-associated metabolic syndrome [103,104].

#### VAT and impaired skeletal muscle oxidation

The amount of fat deposited within skeletal muscle (intramyocellular lipid – IMCL) and the ability of muscle to oxidize fat are important determinants of weight gain,[105] weight regain following weight loss [106], and the development of insulin resistance syndrome [107]. IMCL and the VAT depot might not be independent from each other. Furthermore, the relationship between IMCL and insulin sensitivity is independent of percent total body fat and SCAT but not of VAT [108]. In individuals with type 2 diabetes, among the depots of regional and overall adiposity, VAT was the depot of adipose tissue that was most strongly related to skeletal muscle insulin resistance [109].

Colberg et al studied the fasting patterns of skeletal muscle fatty acid uptake and oxidation in healthy, lean and obese premenopausal women who had a cross-sectional VAT area over a range from 18 to 180 cm^2 ^and BMIs from 19 to 39 kg/m^2 ^[110]. The researchers found that insulin sensitivity as well as postabsorptive rates of FFA utilization or oxidation by muscle were diminished in relation to VAT. Women with increased VAT did not have lower plasma FFA levels or lower rates for appearance of FFA, yet they had an impaired or reduced uptake of plasma FFA by the skeletal muscle in the leg [111]. Together, this supports a role for VAT, IMCL lipid deposition, and perhaps impaired oxidation of nonadipose tissue lipid in insulin resistance and metabolic syndrome.

#### VAT may influence central SCAT

Mauriege et al found that adrenoreceptor sensitivity was increased in SCAT cells of individuals who have a higher VAT accumulation compared to those with a low VAT deposition [112]. SCAT adipocytes from women with visceral obesity exhibit higher lipolysis rates in vitro than those obtained from women with little VAT [113]. Mauriege et al also demonstrated that among men with high levels of VAT, SCAT adipocytes are more sensitive to β-adrenergic lipolysis which may further exacerbate an impaired insulin action, a potentially important factor in the etiology of metabolic syndrome associated with visceral obesity [112]. Moreover, an increased truncal SCAT mass and an increased amount of VAT mass can independently predict insulin resistance [114]. Together, these findings support that VAT may enhance central SCAT lipolysis and accelerate release of peripheral FFAs.

The PPARs are important transcription factors that play an important role in the induction of adipose-specific genes, the proliferation and differentiation of adipocytes, and the development of mature adipose tissue. A number of transcription factors are involved, including PPARγs. Giusti et al suggest that in VAT, the expression of PPARγ2 is controlled by local transcription factors (RXRα, αSREBP1, and SREBP1c) promoting fat storage in adipocytes. Given that the fat storage capacity is limited in VAT, RXRα induces the expression of PPARγ2 in SCAT to increase its overall capacity [115]. These data also suggest that the signal to promote fat storage may occur in VAT and that other metabolic and hormonal factors are involved in the control and modulation of adipogenesis in visceral fat [115].

Perhaps the above can be explained as follows. SCAT cells may act as a buffer or sink for circulating FAs and TGs but once they reach their capacity they lose their protective benefits. Initially, VAT may influence SCAT to expand and act as a buffer. However, once the critical VAT threshold (CVATT) is achieved and metabolic syndrome has begun to develop, then VAT may influence central SCAT to become more VAT-like, i.e., more lipolytic and less sensitive to insulin's adipogenic or lipid storing effects.

### SCAT

#### Greater preadipocyte differentiation and protection

As discussed earlier, preadipocytes from SCAT depots have a greater capacity than VAT to differentiate into numerous, small, insulin-sensitive, adipocytes [70,71]. These lipid-storing cells act as a buffer or sink for circulating FAs and TGs, thereby preventing their deposition in non-adipose tissues, e.g., skeletal muscle, pancreas, and liver, where they could contribute to lipotoxicity, apoptosis, and insulin resistance [73,116].

#### Does SCAT replenish VAT?

In defending the role of VAT accumulation in individuals with metabolic syndrome, we must postulate a high rate of lipid turnover, with high rates of lipolysis at certain times matched by high rates of lipid deposition at other times. Otherwise, as Frayn points out, the hyperlipolytic VAT would ultimately disappear [117]. He also suggests that if SCAT were to become insulin resistant, and therefore resistant to fat storage, then fat might tend to be deposited in VAT depots. Another possibility is that the usually larger SCAT depot has a greater potential to contribute to insulin resistance through release of FFA into the systemic circulation. However, this would not adequately explain the subset of individuals who demonstrate metabolic profiles consistent with insulin resistance but are in fact lean, healthy-appearing with normal BMIs, excess VAT, little SCAT, and are referred to as "metabolically obese, normal weight (MONW) [26]. As described above, perhaps once VAT expands and SCAT depots reach their capacity for storing FAs, then do SCAT adipocytes become insulin resistant, release FFAs, and contribute to systemic insulin resistance and metabolic syndrome.

While some studies cast doubt on the portal theory and its implications for VAT's direct delivery of FFA to the liver [118,119], they leave open other mechanisms via which VAT could induce insulin resistance and other metabolic disturbances, e.g., by producing proinflammatory cytokines which could be directly delivered to the liver where they can potentially affect hepatic metabolism [117]. These will be discussed below.

#### Peripheral fat mass may protect against atherosclerosis and metabolic syndrome

If trunk fat is taken into account, accumulation of fat in the hips and legs is an independent predictor of lower cardiovascular and diabetes-related mortality, and it seems to protect against impaired glucose metabolism, especially in women [120-124]. In a study of 1,356 women ages 60–85, those with excessive peripheral fat had less atherosclerosis (determined by aortic calcification scores), and the quartile with both the highest amount of central fat and peripheral fat seemed to be partially protected by the high percentage of peripheral fat mass as reflected in a number of measured risk factors [121]. These findings corroborate similar findings by the same group who followed 316 postmenopausal women for 7.7 years and monitored progression of aortic calcifications [120]. In yet another study, Tanko et al demonstrated that peripheral fat mass (SCAT) in generally obese, post-menopausal women is associated with increased adiponectin and higher insulin sensitivity [125]. Together, these support protective roles for peripheral fat. In addition to fat trapping, these might include possible influences on adipokines, e.g., they might contribute to an increase in adiponectin, which could improve FA oxidation [126]. One must interpret these results with caution because the measuring technique of dual-energy X-ray absorptiometry (DXA) does not allow separate quantification of intermuscular and subcutaneous fat in the arms and legs as well as SCAT in the trunk [121]. While VAT is a major predictor of insulin sensitivity in overweight and lean individuals [114,127], others have found abdominal SCAT to contribute to insulin resistance independently of VAT [128,129].

#### An example of metabolically innocent obesity

When there is an inability to store fat, due to lipodystrophy, the adipocytes' storage capacity is exceeded and lipids accumulate and cause lipotoxicity in liver, muscle, and other organ tissues [7]. A counterpart of lipodystrophy may be illustrated by patients with multiple symmetric lipomatosis (MSL), a condition characterized by regional excess of subcutaneous adipose tissue. These patients have higher adiponectin levels, a high degree of insulin sensitivity and glucose tolerance, very low lipid levels in liver and muscle cells, and markedly little VAT [130]. In this case, SCAT may be protective and beneficial. This may be analogous to thiazolidinedione action, which also promotes SCAT deposition while improving insulin sensitivity and glucose tolerance [74,75].

#### Estrogen

Estrogen promotes the accumulation of peripheral gluteo-femoral SCAT, which may be protective [131]. The abundant presence of peripheral fat mass in generally obese women is associated with increased plasma adiponectin, and the loss of estrogen with menopause is associated with an increase in central fat [132]. This accounts for the progression in many overweight women after menopause from a predominantly pear-shape or "gynoid" habitus to the apple or "android" shape. Contrary to popular belief, menopause does not seem to independently cause a gain in total body weight; the increases in BMI that often accompany menopause are usually consistent with normal aging [133]. However, even without weight gain, body fat distribution changes; postmenopausal obese women tend to accumulate abdominal fat along with deterioration of risk factors, even if total body weight and BMI do not change during menopause transition.

After menopause, when ovarian function declines, adipocytes become the primary source of endogenous estrogens [134], and compared to "gynoid" or pear-shaped women, those with central obesity (apple- or "android-" shaped) have lower plasma SHBG and higher estradiol [125,135]. This suggests regional differences in the enzymatic conversion of steroid hormones in VAT versus SCAT [125,136-138]. In ovarian hormone-deficient women, SCAT adipocyte size, lipoprotein lipase (LPL) activity, and basal lipolysis were not found to be significantly greater compared to regularly cycling premenopausal women. However, in the ovarian hormone-deficient women, omental (VAT) adipocyte size was significantly higher, and the omental/SCAT LPL activity ratio and VAT lipolysis were also significantly higher [139]. For a given amount of total body fat, men have been found to have about twice the amount of VAT than what is found in premenopausal women but this may change after menopause when VAT storage becomes predominant [140,141].

Along with an increase in VAT, a decline in estrogen is also associated with reduced lean body mass as well as other features of the metabolic syndrome including: dyslipidemia with elevation in Lp(a), triglycerides, and an increase in small, dense, LDL particles. Estrogen deficiency also may influence cardiac risk by its effects on the insulin action, the arterial wall, and fibrinolysis. Park et al showed that postmenopausal women lost less VAT compared with the premenopausal women during a weight reduction program (10.5 percent vs. 25.7 percent respectively) [142]. The reasons behind this are presently unclear.

As mentioned above, in menopause, adipocytes are primary sources of endogenous estrogens in women [125,134], and estrogens are known inhibitors of IL-6 secretion [143]. It is worth noting that the relationship between BMI and serum IL-6 was observed only in postmenopausal women, and this relationship was lost among those women receiving hormone replacement [144]. Adipose tissue-derived estrogens in postmenopausal women would not be sufficient to reduce IL-6 in a similar way as endogenous estrogens do in premenopausal women [145]. Perhaps in premenopausal women, endogenous estrogen from the ovaries helps keep VAT volume relatively low and is thereby protective. Estrogen by itself seems to protect postmenopausal women receiving replacement therapy from VAT accumulation, and in women with type 2 diabetes, estrogen replacement may protect against the risk of cardiac events [146,147].

Compared to men of similar age, premenopausal women appear to be significantly protected from CHD. However, by age 70 the incidence of CHD is equal in men and women, suggesting that estrogen deficiency causes a rapid acceleration in CHD risk [133]. Yet, in elderly, postmenopausal women, Tanko et al showed that those women with higher amounts of central versus peripheral obesity had significantly higher levels of estradiol and lower adiponectin. This suggests that prolonged and increased exposure of SCAT cells to estradiol may eliminate the protective effect of SCAT by affecting SCAT's ability to release adiponectin thereby promoting the atherogenic effects of IL-6 [125]. Perhaps future research will help clarify whether central obesity has any implication for increased susceptibility to the adverse cardiovascular effects of hormone replacement therapy (HRT) in diabetic patients early after initiation of therapy [125].

Obesity, particularly visceral obesity, as well as insulin resistance and hyperinsulinemia are associated with breast cancer [148]. Insulin may increase estrogen action by increasing bioavailable estrogen due to a decrease in sex hormone-binding globulin, by influencing estrogen receptors, and by increasing aromatization of androgen to estrogen at the tissue level, a phenomenon which has been demonstrated in breast tissue. Estrogen upregulates the IGF-1 receptor and IGFBP-1 and -2 and may directly activate the IGF-1 receptor, thereby increasing insulin signaling [149].

Around 1900, most women died soon after menopause. The average lifespan of persons in the United States has since lengthened by greater than 30 years [150], which means that women, and men, too, are now spending 30 or more years with hormonal and physiological states that society and medicine has not had to deal with previously. These, combined with significant dietary and lifestyle changes since 1900, must be considered as critical contributing factors to the world's current epidemic of metabolic syndrome.

### Lipotoxicity Model

#### Overnutrition, lipotoxicity, leptin, and the metabolic syndrome

When one consumes too many calories, especially in the form of excessive carbohydrates, the liver converts excess glucose to fatty acids. First, glucose that is not oxidized or stored as glycogen is metabolized to acetyl CoA, which then enters the lipogenic pathway. Acetyl CoA is catalyzed to form malonyl CoA, which in turn inhibits carnitine palmitoyl transferase 1 (CPT-1, the enzyme responsible for fatty acid transport into the mitochondria) [42]. The net effect is that malonyl CoA (from excess carbohydrates, glucose, and insulin) reduces the oxidation of FAs [151]. This results in increased accumulation of intracellular fat in the form of long chain fatty acids and their derivatives, e.g., TGs and ceramide [28,29]. Cellular TG accumulation is not initially toxic and may actually be protective by diverting excess FAs from pathways that lead to cytotoxicity [152]. While glucose is being preferentially utilized, the FAs are metabolized by pathways other than their preferred β oxidation, leading to toxic products, e.g., ceramide, which may cause apoptosis and lipotoxicity [28,30,153]. The subsequent development of the cell's resistance to insulin-mediated glucose uptake, which prevents further influx of glucose, may be viewed as being protective in that it limits the amount of intracellular glucose to be preferentially metabolized over the β oxidation of intracellular FAs [29,37,154]. The cell can be insulin resistant with respect to glucose uptake and metabolism but remain sensitive to insulin's lipogenic effects and the de novo synthesis of fat. Overconsumption of calories, especially in the form of carbohydrates, also stimulates hyperinsulinemia that can then upregulate SREBP-1c and increase de novo lipogenesis [43].

### Leptin protects against lipotoxicity

#### Leptin

The first adipocyte-specific hormone to be characterized, leptin is produced predominantly by SCAT adipocytes compared to VAT. Females produce leptin at about twice the rate in males [155], and leptin secretion increases with enlarged adipocyte cell size. Circulating leptin rises by 40 percent after acute overfeeding and more than three-fold after chronic overfeeding, whereas fasting is associated with decreased leptin levels [156].

#### Dietary carbohydrates may influence leptin action

The increase in leptin concentration after meals is not simply a result of a caloric load, but is in response to a signal that is not present following a fat load without carbohydrate [157]. Leptin circulates in a free form and is also bound to a soluble leptin receptor – sOBR, which is positively associated with energy intake from carbohydrates and negatively associated with energy intake from dietary fat [158].

Excess caloric consumption and fat deposition results in newly synthesized FAs that are transported as VLDLs and stored as TG in adipocytes. Initially, these expanding adipocytes secrete leptin in proportion to their growing fat accumulation. Leptin also crosses the blood brain barrier, stimulates its receptor in the hypothalamus, and causes the release of neuropeptide-Y (NP-Y), which reduces feeding behavior [85]. This, in turn, suppresses appetite and stimulates thyroid function. Leptin affects peripheral tissues, and is a determinant of insulin sensitivity. The ensuing hyperleptinemia increases fat oxidation in skeletal muscle [159-161], and also keeps de novo lipogenesis in check by lowering the involved transcription factor, i.e., SREBP-1c mRNA (sterol regulatory element binding protein 1c mRNA) [43]. It promotes cholesterol ester synthesis in macrophages in a hyperglycemic environment, an important process in the formation of foam cells in atherosclerosis which may suggest a protective role of relative leptin resistance [162]. Leptin also possibly increases sympathetic nervous system (SNS) activity with subsequent decreased FFA oxidation and thermogenesis [163]. All of these effects of leptin tend to limit further weight gain.

### Leptin resistance

As the process progresses, inefficient leptin action can lead to the opposite of leptin's protective effects, e.g., hyperphagia, decreased fat oxidation, increased tissue TG levels, insulin resistance, and overweight. Subsequently, plasma leptin levels rise. The majority of obese individuals with high leptin levels show a leptin insensitivity or "resistance [164]," which occurs at the leptin receptor level. In animal models, leptin-resistance and leptin-deficiency increases, and upregulates the hepatic expression of SREBP-1c mRNA, which may stimulate an increase in fat production via de novo lipogenesis. Together, all of these features suggest a state of "leptin resistance" which may ultimately lead to obesity and metabolic syndrome [29,165].

It is quite possible that hyperleptinemia in diet-induced obesity serves to protect nonadipose tissues (e.g. muscles, liver, pancreatic β cells, and myocardium) from the toxic effects resulting from the spillover of full adipose stores and subsequent ectopic deposition of FFAs. In defense of this paradigm, Unger points out that normally rats can tolerate a 60 percent fat diet because 96 percent of the surplus fat is stored in an enlarging adipose tissue mass, in which leptin gene expression increases proportionally [166]. However, when leptin is congenitally absent or inactive, or ineffective due to resistance, even on a normal or low-fat diet, excess dietary fat is deposited in nonadipose tissues. This causes dysfunction (lipotoxicity), and possible cell death (lipoaptosis) [29].

Acquired leptin resistance occurs in aging, obesity, Cushing's syndrome, and acquired lipodystrophy, a condition associated with protease inhibitor therapy of AIDS. Preliminary evidence suggests that patients with these conditions have increased ectopic fat, i.e., lipid deposition in non-adipose tissues [29].

#### Role of triglycerides in leptin resistance

The relation between cerebrospinal fluid and serum levels of leptin in obese humans suggests that defective blood brain barrier (BBB) transport accounts for a great deal of leptin resistance in the CNS. Banks et al showed in mice that serum TGs directly inhibit the transport of leptin across the BBB and so could be a major cause of leptin resistance across the central nervous system (CNS). Thus they suggest that serum TGs are likely a major cause of the leptin resistance seen in both obesity and starvation [167]. This hypothesis explains why lowering TGs may be therapeutically useful in enhancing the effects of leptin.

#### Implications for VAT in relative hypoleptinemia and metabolic syndrome

Compared to VAT, SCAT is the predominant source of leptin [60], yet patients with VAT obesity may tend to have higher leptin levels than normal, lean individuals but lower than those with predominantly SCAT or subcutaneous obesity [29]. This suggests that the hyperleptinemia of predominantly VAT obesity is not high enough to overcome a leptin resistance due to the accumulation of ectopic fat in nonadipose tissues, which leads to lipotoxicity and ultimately the metabolic syndrome [29].

#### Lipodystrophies – A paradigm for the roles of fat depots and insufficient leptin action in metabolic syndrome

A number of clinical states exhibit evidence of leptin insufficiency, either leptin deficiency or resistance, and they all have in common the metabolic syndrome. These include rare genetic diseases known as lipodystrophies, which are characterized by a redistribution of fat. Ironically, in the more severe cases, e.g., congenital generalized lipoatrophy, near-complete fat loss is associated with severe insulin resistance, fatty liver, and classic features of the metabolic syndrome. There is hyperleptinemia along with hyperphagia and a predominance of intra-muscular fat [168]. Dunnigan-type familial partial lipodystrophy is a rare autosomal dominant condition characterized by markedly reduced plasma leptin levels along with gradual loss of SCAT from the extremities, trunk, and gluteal region, commencing at the time of puberty, as well as hyperinsulinemia, glucose intolerance, dyslipidemia (high TGs with low HDL), and diabetes [169,170]. These individuals do maintain central obesity and VAT [169], which supports a relatively protective role for SCAT and implicates VAT as being more pathogenic.

The aforementioned potential role of TGs in leptin resistance may have implications for patients with lipodystrophy and lipoatrophy who have little or no fat mass, and as a result, have very little or no leptin. They also have severe hypertriglyceridemia that is reversed by treatment with leptin [168,171]. The elevated plasma level of TGs in these patients is likely inducing leptin resistance that is preventing the leptin from inducing TGs to be used as an energy source. Thus the TGs in these patients are not oxidized, and they are unable to settle into fat stores that would normally act as a TG sink and prevent their diversion to non-adipose tissues where they contribute to lipotoxicity and insulin resistance.

### Fat depots can protect against lipotoxicity

#### Fat provides leptin and adiponectin

Transplantation of adipose tissue grafts in animal models of congenital lipoatrophy reverses the signs of the metabolic syndrome in a dose-dependent fashion [172]. Furthermore, leptin treatment in humans and animals with lipodystrophies also reverses fatty liver and insulin resistance. However, transplantation of ob/ob adipose tissue (which does not produce leptin) in lipodystrophic rats does not reverse diabetes [173] nor is it beneficial to inject leptin in obese humans with leptin resistance [18]. These support the notion that insufficient leptin action may be a cause of metabolic syndrome, and that adequate leptin derived from SCAT is protective.

Like leptin, adiponectin secretion increases early on in obesity and plays a role in reducing the expression of lipogenic enzymes and increases FA oxidation in peripheral tissues thus limiting ectopic fat accumulation [174]. The fact that adiponectin is secreted initially by fat but levels are reduced as fat depots increase, may help resolve the paradox of both lipodystrophy and obesity both being insulin-resistant states [73].

### Critical Visceral Adipose Tissue Threshold (CVATT) – Individual Variation

The CVATT has tremendous individual variation; thus a relatively "thin" individual (with a normal BMI) and an excess of VAT for him, may be metabolically obese, normal weight (MONW) [26]. Meanwhile, another individual with a large "pot belly" may have a great capacity to store fat as SCAT with relatively little VAT or he may have a high threshold for VAT. This may explain the finding that while some individuals weighing even up to 200 kg do not show any signs of type 2 diabetes or dyslipidemia, while in others, diabetes or dyslipidemia either develop or deteriorate with an increase in body weight of only one kg [175] – perhaps just enough to exceed the CVATT.

A number of studies have looked at a possible CVATT [176-182]. Using CT scans to measure VAT volume, Williams et al found that a value of above 110 cm^2 ^was associated with an increased risk of CHD in pre-and postmenopausal women [177]. Similarly, Despres and Lamarche observed a VAT cutoff of 100 cm^2 ^was associated with increased CHD risk in young adult men and premenopausal women (mostly of French Canadian descent) [179], and a cutoff range of 100–110 cm^2 ^has also been observed by others [176,181]. Other studies have suggested thresholds of > 130 cm^2 ^for metabolic deterioration [183,184]. De Nino et al found that insulin resistance did not appear until women were older than 60 years and had accumulated levels of VAT that approximated the levels seen in men, suggesting a possible threshold effect of VAT on insulin resistance [185]. As discussed below with MONW, genetic and ethnic factors play a role. For example, in nonobese and obese Japanese males and females, fat areas at the umbilicus as determined by CT had threshold values for metabolic syndrome with only > 100 cm^2 ^for men and > 90 cm^2 ^for women [186].

Brochu et al were unable to demonstrate that obese postmenopausal women who reduced their weight and attained a level of VAT below 110 cm^2 ^would show greater improvement in their metabolic profile compared to those who also lost weight but remained above the 110 cm^2 ^VAT threshold [178]. However, there were only 25 total subjects and the women had relatively normal metabolic profiles at baseline. Perhaps due to the relatively small number of subjects, only five lost less than 20 percent of their baseline VAT value. Thus it is unclear whether even smaller losses of VAT than those observed improved metabolic outcomes. The researchers did find larger losses of VAT and a greater improvement in insulin sensitivity in those who attained a VAT level < 110 cm^2 ^[178]. It should also be noted that in postmenopausal women, peripheral SCAT may be protective, even in the face of large amounts of VAT, and this needs to be accounted for [120,121,125]. While studying obese Japanese women, Tanaka et al recently validated the 100 cm^2 ^CVATT but their longitudinal data from both pre- and posttreatment suggest that these women should reduce their VAT area to <60 cm^2 ^through weight reduction to improve CHD risk factors [181].

### Metabolically obese normal weight (MONW)

VAT accumulation contributes to metabolic risk factors in nonobese individuals [187,188]. Ruderman et al have shown that normal weight individuals may also have insulin resistance and the disorders of the metabolic syndrome [26]. They designated such individuals as "metabolically obese normal weight – MONW [189,190]." MONW subjects (BMI < 25 kg/m2) have been characterized by an excess of VAT area (> 100 cm^2 ^by abdominal CT), insulin resistance, and hyperinsulinemia [24-26]. As pointed out earlier, the development of insulin resistance may limit further weight gain [34,38-41,191]. A rapid and early development of insulin resistance prior to significant weight gain would explain that a significant number of the normal-weight population have insulin resistance [26]. The prevalence of MONW could be as high as 13 – 18 percent [26,192,193].

#### MONW and low birthweight

Both low birthweight (LBW) [194] and lowest weight at one year of age have been linked to, VAT accumulation[195] insulin resistance and cardiovascular risk factors in middle-aged and elderly individuals, many of whom could be classified as MONW with metabolic syndrome. By middle age, many LBW subjects have BMIs less than 24–26 kg/m2 and would be classified as MONW. While some data suggest that LBW babies have central adiposity in middle age, definitive measurements of VAT in these individuals are still lacking [26].

#### Ethnicity and MONW

One should consider ethnic differences when attempting to identify MONW subjects. Lean appearing individuals, especially in certain ethnic groups such as the Japanese, may have significant amounts of VAT that surpass their CVATT but appear with what, for the general population, would be considered a normal BMI and waist circumference [196]. For example, nonobese Japanese (BMI<25) with increased VAT areas (100–110 cm^2^) fulfill the criteria for MONW [25,181]. In another study, relatively lean Japanese patients with newly diagnosed type 2 diabetes had increased VAT. Through diet and without medication for three months, the amount of VAT in these patients became comparable to that in normal-weight control subjects. Therefore, a three-month dietary treatment regimen with small to moderate weight loss was very effective in decreasing excess VAT in this population [197]. This illustrates the importance of early recognition of an individual's approaching or exceeding his CVATT. Park et al were among the first to demonstrate that healthy, non-obese Asian American women may have higher amounts of VAT, and that normative values or standards for VAT derived from Caucasians may not be applicable to Asians [196]. On the other side of the spectrum, a 10-year prospective study studied increased BMI in Micronesian Nauruans (an ethnic group from the central Pacific Ocean with rapidly increase in prevalence of obesity) and Melanesian- and Indian-Fijians. Overall, there was little evidence to suggest that obesity was a risk factor for total or cardiovascular mortality in these populations [198].

### Metabolically normal obese (MNO)

McGarry found that one of his most obese patients in a series (BMI 32.8 kg/m2) was one of the most insulin-sensitive but had one of the lowest values for intramyocellular lipid (IMCL). Conversely, another subject, with a BMI of only 18.9 kg/m2, proved to be highly insulin-resistant but had a large amount of IMCL. This supports that insulin sensitivity appears to correlate more with where the fat is located rather than the total amount in the body [42]. This has implications for the phenomena of the metabolically obese normal weight (MONW) and the metabolically normal obese (MNO) individuals.

Like some of the Micronesian Nauruans and Indian-Fijians above, there are individuals who are obese and who nevertheless are metabolically normal – "metabolically normal obese; MNO." Unlike their MONW counterparts, MNO individuals have very little VAT accumulation. They often share an onset of obesity early in childhood, normal VAT, lower TGs, and increased HDL. The actively competitive Japanese wrestlers maintain their gross obesity by consuming a 5,000 to 6,000 calorie diet. They are MNO, and their VAT is normal in amount, i.e., they have excessive amounts of SCAT [91]. On retirement, when they discontinue their rigorous training regimen, they markedly develop increased insulin resistance and metabolic syndrome. It is likely that that their VAT increases concomitantly [26,27] and exceeds their CVATT. Data from the European Group for the Study of Insulin Resistance (1146 hyperinslinemic/euglycemic clamp studies from 20 clinical centers in Europe) showed that in "simple" obesity, insulin resistance is not as prevalent as previously thought [199]. MNO could account for as much as 20 percent of the obese population [193]. In another study using HOMA to determine insulin resistance, Bonora et al showed that 11 percent of the entire group of overweight individuals fit the criteria of MNO [200]. Brochu et al extensively studied 43 sedentary, obese, postmenopausal women and found that 17 were MNO, while 26 had reduced insulin sensitivity (estimated by clamp) [201]. The two groups were similar in total body fat mass, SCAT amount, as well as waist circumference, and total daily energy expenditure. However, lean body mass was significantly greater in the metabolically abnormal subjects. Unlike SCAT, VAT measured by CT was inversely related to the insulin sensitivity and to a classification of MNO. In fact, despite similar levels of total body fatness, MNO individuals showed 49 percent less VAT as measured by CT. However, the level of VAT was still significant. Furthermore, using doubly labeled water and indirect calorimetry, Brochu et al were unable to demonstrate a meaningful difference between resting metabolic rate and daily physical energy expenditure between MNO and obese individuals at risk [201].

#### MNO and childhood obesity

Several investigators have found that there has been a positive association between insulin sensitivity and duration of obesity, i.e., those who are obese since childhood are more likely to remain insulin sensitive. In one study 48 percent of the MNO women presented with a history of an earlier age-related onset of obesity (between 13 and 19 years of age) and less VAT compared with 29 percent of the metabolically abnormal obese [201].

Insulin sensitivity seems to be dependent upon adipose cell size; as adipocytes within tissue grow larger, they become more insulin resistant [202]. Normal-sized, more insulin-sensitive adipocytes have been associated with early onset of obesity [203]. Perhaps today we are beginning to see that with the marked increase in overfeeding and extent of obesity at younger ages, hypertrophy of fat cells may occur earlier and hence metabolic syndrome is now occurring with greater frequency in children.

### Puberty and VAT

During puberty, a certain degree of insulin resistance is normal, and children who are more insulin resistant have decreased SCAT fat gain [204]. Early in the development of juvenile obesity, increased VAT, hyperinsulinemia, and insulin resistance are closely linked [205]. Adrenal androgens are elevated in obese children and have been associated with early pubertal development in these children[206,207] Sex differences in VAT begin to emerge during puberty, with boys having more VAT than girls. Some studies suggest that the rate of VAT accumulation can be slowed in children by using exercise interventions [208,209].

### Fit and fat

VAT is strongly associated with fitness even within individuals of the same weight. This is illustrated by the earlier mentioned example of the active Sumo wrestler in his prime who has relatively little VAT [91]. Regular exercise can selectively reduce VAT with minimal change in weight [210-212]. This could especially add to the frustration level of the middle-aged or post-menopausal woman who regularly exercises moderately without inducing measurable reduction in body weight or fatness. She may still benefit from reducing her VAT or attenuating the gain of VAT "normally" experienced by sedentary women as they age. It should be emphasized that the lower VAT level associated with increased fitness is modest but nonetheless clinically important. Reduced morbidity is likely explained by factors in addition to a reduced VAT, and VAT likely explains morbidity independent of fitness [213]. Sumo wrestlers tend to have most of their central adiposity stored subcutaneously (as SCAT), and, perhaps a shift toward more VAT accompanies their contracting metabolic syndrome upon their retirement – with premature death to follow [26,91]. This may also explain the body of work showing that overweight or "fat" individuals who are fit (according to cardiorespiratory testing on a treadmill) are at less risk for a cardiac event or developing type 2 diabetes than a "leaner" individual who is unfit [214,215]. Thus, the former could be considered "fit and fat." High levels of cardiorespiratory fitness (CRF) reduce CRP and the rate of cardiovascular morbidity and mortality, independent of obesity [216]. CRF is also associated with lower abdominal fat independent of BMI, and for a given BMI or waist circumference (WC), individuals with moderate CRF had lower levels of total fat mass and abdominal SCAT and VAT than individuals with low CRF for a given BMI or WC value [213,217]. Low CRF is an independent risk factor for mortality in healthy-appearing and diseased populations, and is associated with elevated CRP and reduced fasting glucose control in women with type 2 diabetes [218]. It is likely that compared to the fit and fat, the unfit and lean-appearing individual may have greater amounts of "hidden" VAT.

#### Effects of exercise

In obese patients, increasing physical activity can enhance fat oxidation, reduce IMCL and improve insulin sensitivity [219]. Exercise training may reduce waist size, independent of changes in BMI, and exercise without weight loss is effective in reducing VAT and preventing further increases in obesity [213,220].

Ross et al showed that either modality, caloric restriction alone or daily exercise without calorie restriction, is an effective strategy for reducing obesity in moderately obese men.

Their findings also suggest that exercise without weight loss is a useful method for reducing VAT and preventing further increases in obesity [220]. Irwin et al studied 168 overweight, postmenopausal, previously sedentary women in a randomly controlled trial of exercise versus no exercise. While the body weight lost at 12 months among the exercisers was modest, the amount of intra-abdominal fat lost was considerable (8.5 g/ cm^2^) and was dose-dependent. The women who exercised for approximately 200 min/wk lost 4.2 percent of total body fat and 6.9 percent of VAT without reducing their energy intake [212]. Exercise may counteract the abnormal metabolic profiles associated with abdominal obesity by reducing VAT along with other independent mechanisms. It promotes adaptive responses including those causing muscles to increase their use of lipid stores rather than relying primarily on carbohydrate reserves. Even a single bout of exercise can reduce triglyceride levels, increase HDL levels, reduce resting blood pressure, increase glucose tolerance, and reduce insulin resistance [221].

While evidence supports that CRF may be associated with a lower VAT, this is certainly not proven. However, study results suggest that individuals with moderate to high CRF levels have lower WC than men with low CRF independent of BMI [213,222]. Data support that the substantial reductions in health risk often associated with modest weight loss (<10 percent) may be mediated in part by a preferential reduction in VAT [48,216,217,220,223]. This is reinforced by the finding that reductions in VAT alone were related to improvements in glucose tolerance and insulin sensitivity [220,224]. Therefore, it would seem reasonable to infer that the combination of high CRF and low abdominal fat, especially VAT, would be associated with reductions in metabolic risk compared with those with the same BMI, but low CRF and high VAT [213]. Adding resistance training to aerobic exercise may add to an improvement in insulin sensitivity related to a loss of VAT and an increase in muscle density [225,226].

### Surgical Interventions Shed Light on Pathophysiology

Surgical removal of VAT in animals and humans dramatically improves insulin resistance and diabetes. In a Swedish, single-center, randomized and controlled pilot trial of 50 severely obese adults, Thorne et al compared 25 patients who underwent adjustable gastric banding (AGB) alone with AGB plus surgical removal of the total greater omentum. At two-year follow-up there were no statistical differences between groups with regard to weight loss, changes in WHR or sagittal diameter. However, the improvements in oral glucose tolerance insulin sensitivity and fasting plasma glucose and insulin were 2–3 times greater in omentectomized as compared to control subjects, which was statistically independent of the loss in BMI [52]. More recently, this has led to a study of another experimental procedure performed by surgeons at Boston's Beth Israel Deaconess Medical Center working in conjunction with Joslin Diabetes Center. Using a two-hour laparoscopic procedure that involves extracting strips of only the omentum through tiny incisions, this will be the first study to examine the possible health benefits of removing only the omentum [227].

Recently Klein et al. demonstrated that liposuction conferred no benefits with regard to metabolic profile [53]. Furthermore, Weber et al showed that after 3 months, animals that had lipectomy of > 50 percent of SCAT had more intra-abdominal VAT as percentage of total body fat, higher insulinemic index, a strong trend toward increased liver fat content, and markedly elevated serum TGs compared with animals that had undergone a sham operation and received either a high- or low-fat diet [228]. Together with the findings above, these support a pathologic role for VAT and a possible protective role for SCAT. Removing SCAT might actually increase risk as one removes a buffer or sink for peripheral TGs [228].

### Environmental Considerations

#### Organochlorines, adipose tissue, and energy balance

Since our genes have not changed significantly in the past 10,000 years, the rise in obesity can be attributed to the environment, including what we are exposed to in the way of food as well as the level of physical activity. While the main focus has been on diet and activity, what may be overlooked is the tremendous increase in exposure to synthetic organic and inorganic chemicals, which can damage many of the mechanisms involved in weight control. Most of us have been exposed to organochlorines found in pesticides, dyes, solvents, etc., and we contain residues in our adipose tissue, where they are preferentially stored. Thus, the obese tend to have increased organochlorine concentrations compared to lean individuals [229]. During body weight loss, a decrease in fat mass results in lipid mobilization, and organochlorine concentrations increase both in plasma and remaining adipose tissue. Even after adjustment for weight loss, the related increase in organochlorine concentration has been correlated with decreases in triidothyronine (T3) concentration and resting metabolic rate [230]. This is also associated with a reduction in activity of the skeletal muscle oxidative enzymes that normally are involved in fat oxidation [231]. The net effect could prevent further weight gain and might even encourage weight regain beyond the initial baseline [232], which could contribute to VAT.

### Implications of Controlling Dietary Carbohydrates

#### Reduced fat oxidation and carbohydrates

Frisancho points out that an important contributing factor for obesity in modern as well as developing nations is a reduced fat oxidation and increased metabolism of carbohydrate. This has been brought about by a shift toward the body's preference toward oxidizing carbohydrate rather than fat – resulting in an increased deposition of body fat. In developing nations, obesity can co-exist with developmental undernutrition, which can result in obesity with short stature [233].

A solution to reducing the ectopic fat, as well as VAT, burden would be to enhance its oxidation in nonadipose tissues, e.g., liver, pancreas, and skeletal muscle. This will push the system toward below the CVATT and improve insulin sensitivity. In their review, Westman et al cite many studies that have consistently shown that low-carbohydrate/high-fat diets consumed for more than seven days induce powerful metabolic adaptations to enhance fat oxidation [37]. Such diets will reduce muscle glycogen content and carbohydrate oxidation, even in well-trained athletes who already demonstrate increased oxidation [37,154]. The authors' paradigm suggests that, under these conditions, insulin resistance could improve by reducing glucose appearance and cellular influx, resulting in a preferential fat oxidation and protection against lipotoxicity. In an elegant study, Bisschop et al support this by showing that high-fat, low-carbohydrate diets do not affect the action of insulin on total glucose disposal but decrease basal endogenous glucose production and improve insulin-stimulated nonoxidative glucose disposal [234]. Sharman et al demonstrated short term improvements of a ketogenic diet on lipids in normal weight men. These benefits occurred without total weight loss but there was evidence of a change in body composition toward more lean body mass [235]. One would also expect a reduction in VAT as he moves to the left or below his CVATT (See figure 1). Weight loss does not appear to be necessary to reduce mortality rates in overweight or obese men who increase their aerobic fitness or level of physical activity [224]. Similarly, in overweight, postmenopausal women, exercise may lead to improved metabolic profiles and VAT loss without total weight loss [212].

### Dietary carbohydrate and VAT

Optimizing macronutrients and food preparation can have beneficial effects in those with visceral obesity. A number of recent reviews support the metabolic benefits of controlling glycemic index (GI) [236] and glycemic load (GL) [237]. In a 12-month pilot study of teens, compared to a conventional diet, a lower GI diet led to greater total weight and fat loss without regain from months 6–12. While insulin resistance as measured by HOMA increased in the conventional group (possibly in part attributable to puberty), the lower GI group showed no change [238]. Recently, Silvestre et al showed that compared to an energy-restricted low-fat diet, a short-term, very low-carbohydrate diet was associated with greater weight and fat loss with an apparent preferential loss of central fat [239].

VAT cells have a two-fold higher glucose uptake rate compared with SCAT cells [87]. It may follow that reducing glucose exposure by reducing glycemic load may reduce the supply of glucose to the VAT depot and possibly impair its accumulation. Glucose raises insulin concentration, which can stimulate 11-β-HSD1, increase active cortisol in VAT, and enhance VAT accumulation [102]. Feeding rats a high-GI starch diet over five weeks resulted in higher VAT and larger adipocyte volume than did feeding low-GI starch ad libitum. Replacing this with a low-GI starch diet increased insulin -stimulated glucose oxidation, decreased glucose incorporation into total lipids and decreased VAT adipocyte diameter [240,241]. Together, these add to the evidence supporting the benefits of lowering GI to reduce and maintain lower volumes of VAT. Feeding rats a high sucrose diet increases both VAT and muscle insulin resistance [242]. Keno et al. demonstrated in rats that a high sucrose diet compared to a lab chow diet led to a significantly greater fat cell volume in VAT depots [243]. Although fat cell number did not change, the ratio of VAT weight to SCAT weight was also significantly increased in the rats fed a high sucrose diet, providing further evidence for controlling the dietary GI and GL.

A number of studies have demonstrated an association between glycemic load (GL) and levels of CRP [244,245], which is a powerful predictor for diabetes and CHD, and is positively associated with both insulin resistance and the prevalence of the metabolic syndrome [246]. O'Brien et al showed that compared to a high carbohydrate diet, a low carbohydrate diet reduced SAA and CRP, both markers of inflammation and risk factors for metabolic syndrome [247]. Relative to fat (cream) and protein (casein), a glucose challenge elicits the greatest production of radical oxygen species (ROS) by polymorphonuclear and mononuclear white cells [248,249]. Chronic carbohydrate ingestion with a high GL diet can lead to hyperinsulinemia, as well as hypertrophy, functional dysregulation, and overresponsiveness of the pancreatic β cell and hepatic production of newly synthesized fatty acids via de novo lipogenesis [43]. A Johns Hopkins study examined intra-operative liver biopsies obtained from 74 consecutive morbidly obese patients undergoing bariatric surgery. Compared with patients with the lowest carbohydrate intake [246], a high-carbohydrate diet was associated with an odds ratio of 7.0 for liver inflammation. A high fat diet appeared to be protective, with those in the highest fat intake group having an OR of 0.17 [250]. This is consistent with the findings of others who found that dietary fat explained only two percent of the variance in general adiposity and dietary fat appears to play only a minor role in determining general adiposity and is not related to VAT when measured in cross-sectional studies [251]. Apparently, GL may be more significant in this regard.

Compared to SCAT, VAT (both adipose and non-adipose cells within VAT) is associated more with PAI-1 – a powerful risk factor for CHD [58,252]. In patients with type 2 diabetes, a simple and modest lowering of the GI compared to an otherwise similar diet led to dramatic changes: a normalized PAI-1 activity (-54 percent, P < 0.001) as well as lowering of both blood glucose and plasma insulin concentrations by 30 percent, and a 29 percent decrease in LDL-C [253]. All subjects began with a BMI < 27, and there was only a slight but similar weight loss in both groups over the 24 days. The results support the potential benefit of lowering dietary GI in patients with metabolic syndrome, especially those with VAT and elevated PAI-1. This is also supported by the observation of hyperglycemia induces PAI-1 gene expression in adipose tissue of rats [254].

Esposito et al demonstrated in both diabetics and non-diabetics that after consuming a high carbohydrate high-fiber meal, IL-18 (a potent pro-inflammatory cytokine) concentrations increased [49]. Adiponectin concentrations decreased after the high-carbohydrate, low-fiber meal in diabetic patients. The fiber content of complex carbohydrates seemed to affect circulating IL-18 and adiponectin concentrations in response to the same carbohydrate load. The pizza that was made with whole flour and was rich in fiber was associated with reduce serum IL-18 concentrations and unchanged serum adiponectin concentrations. Meanwhile, the pizza prepared with refined flour and was low in fiber raised circulating IL-18 concentrations. Serum glucose and TG concentrations were not significantly different between the two types of pizza. The study did not completely resolve the mechanism by which the fiber content of meals influences IL-18 and adiponectin. However, it appears that while the GL of each pizza was the same, the GI of the whole wheat pizza would be much less and may be more beneficial.

Recently, dietary TGs have been demonstrated to contribute to CNS leptin resistance by impairing the transport of leptin across the blood brain barrier where it would usually stimulate the release of neuropeptide-Y and reduce feeding behavior [167]. Reducing dietary carbohydrates lowers serum TGs, which theoretically should protect against this form of leptin resistance [167].

### Dietary influences on leptin action

Leptin may enhance fatty acid oxidation and protects against fat deposition and lipotoxicity. As mentioned earlier, normally, rats can tolerate a 60 percent fat diet because 96 percent of the surplus fat is stored in an enlarging adipose tissue mass, in which leptin gene expression increases proportionally [166]. However, when leptin is congenitally absent or inactive, or ineffective due to resistance, even on a normal or low-fat diet, unutilized dietary fat is deposited in nonadipose tissues, causing dysfunction (lipotoxicity), and possible cell death (lipoaptosis) [29].

Acute overfeeding can cause circulating leptin levels to rise by 40 percent and more than three-fold after chronic overfeeding, whereas fasting is associated with a decreased leptin levels. This may suggest that overfeeding leads to leptin resistance. Dietary carbohydrates may influence leptin action. Some investigators have suggested that the increase in plasma leptin concentration observed after meals is not simply a result of an energy load but is in response to a signal that is not present following a fat load without carbohydrate [157]. SCAT-derived leptin (which circulates in a free form and is bound to a soluble leptin receptor – sOB-R) plays a key role in regulating energy homeostasis and metabolism, sOB-R is positively associated with energy intake from carbohydrates and negatively associated with energy intake from dietary fat [158]. While this suggests that dietary fat and carbohydrates regulate free leptin levels, the implications of this are not yet completely clear.

### Stress

There is an association with lifestyle, worry, cortisol levels, and abdominal girth. Those who were found to have the highest levels of chronic stress had the highest levels of cortisol and VAT [255-257]. This is supported by evidence that a number of medications, including prednisone, may cause an excess of cortisol and insulin resistance. Taken orally, cortisol raises blood pressure, and it has been shown to impair brachial artery blood flow in response to an acetylcholine challenge, i.e., an indicator of endothelial dysfunction [88,255,257-262]. Even brief episodes of mental stress, such as those encountered in daily life, may cause transient endothelial dysfunction even in young, healthy individuals ([263,264]. In turn, subsequent cytokine release may increase anxiety and have negative effects on emotional and memory functions [265]. Psychological stress has also been demonstrated to acutely reduce clearance of triglycerides [266], which could contribute to CNS leptin resistance [167].

There are many other ways in which psychological stress might increase the likelihood of developing metabolic syndrome and type 2 diabetes, for example, chronic psychological stress may also be related to central activation of the HPA (hypothalamo-pituitary-adrenal) axis and the sympathetic nervous system (SNS) [267]. Psychological stress also induces IL-6, TNFα, and other cytokine secretions from macrophages [267-271]. Repeated stress with the repeated induction of corticosteroids can damage the hippocampus, which is involved in the downregulation of corticosteroid production by corticosteroid feedback. Impairment of this feedback mechanism can lead to persisting elevated circulating cortisol levels [267], which might play a role in inducing VAT accumulation.

Stress decreases splanchnic blood flow, impairs the integrity of the GI tract, increases intestinal permeability, and results in increased absorption of lipopolysaccharide endotoxin (LPS) from the gut (the greatest source of LPS). Elevated portal bloodstream LPS levels stimulate Kupffer cell receptors and cytokine release and possibly other immune-challenging activators, e.g., AGEs in food [271].

### Stress and dietary carbohydrates

Dietary carbohydrate has been known to stimulate SNS activity though a number of studies have emphasized the role of insulin. Recent studies in rats have demonstrated that adding glucose to the basic diet increased SNS activity in peripheral tissues and increased GLUT 4 activity in interscapular brown adipose tissue and retroperitoneal fat (but not in epididymal fat) [272]. Overfeeding results in high insulin levels. In the presence of glucose, insulin acts on the brain to increase the SNS tone, which, in turn enhances thermogenesis and dissipation of excess calories [163]. There is a close relationship between postprandial insulinemia, SNS activation, and adipose tissue blood flow (ATBF). ATBF increases in response to stress states such as exercise or mental stress, and also in response to nutrient intake [273]. High insulin levels and increased SNS tone are useful for the maintenance of caloric balance, but in the long term they are conducive to CHD, hypertension, sudden death, and obesity as the SNS receptors become down regulated [163].

Chronic stress leads to elevated cortisol levels, which may lead to accumulation of VAT and metabolic syndrome [274]. Stress-induced increased levels of glucocorticoids can also have a major effect on food intake [275]. A subset of stressed or depressed humans may overeat, especially comfort food (e.g., sugar and fat), in an attempt to reduce anxiety and activity in the chronic stress-response network. This is supported by the finding that these people have decreased cerebrospinal corticosteroid releasing factor, catecholamine concentrations, and HPA activity. While comfort foods may calm them down in the short term, they may lead to abdominal obesity if this becomes a long term "solution." The chronic elevation of systemic glucocorticoids may contribute to VAT deposition. By itself, being obese may be a stressful stimulus to overeating. A weight loss program can be stressful, which can sabotage its success by eliciting the release of stress hormones, which, in turn can make a person crave high energy foods [275]. Feeding rats a long-term high-sucrose diet along with supplemental dexamethasone has been shown to increase fat depots and induce liver steatosis [276]. In addition to dietary intervention, stress management may improve one's cognitive, behavioral, and physiologic responses to stress, including glycemia [277].

## Summary

**Figure 3:**
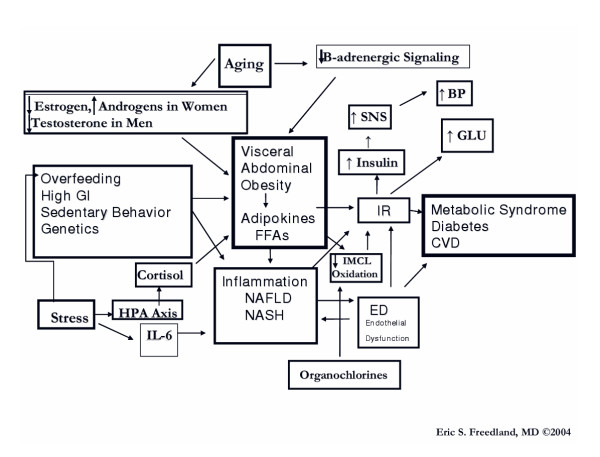
This diagram provides an overview of how behavioral and environmental factors can lead to VAT obesity and, ultimately, metabolic syndrome and related disorders.

The role of visceral adipose tissue (VAT) obesity in metabolic syndrome is critical and complex. (See figure 3). The paradigm of an individual critical VAT threshold (CVATT) has been presented along with a review of potential mechanisms and contributing factors. This includes the potential role of dietary carbohydrates in VAT obesity. As this area continues to evolve, perhaps the reviewed material and proposed concepts may have relevance to clinical assessment, prevention, and treatment of metabolic syndrome.

## Declaration of Competing Interests

The author(s) declare that they have no competing interests.
